# Trends of organ donation and awareness in Ernakulam, Kerala

**DOI:** 10.1186/1753-6561-6-S4-P48

**Published:** 2012-07-09

**Authors:** K Guleria, AK Singh, B Kumar, P Agrawal, S Agrawal

**Affiliations:** 1 Amrita Vishwa Vidyapeetham, India

## Introduction

This study was conducted to get an insight into the knowledge, attitute and practice of the urban and rural poppulations of Ernakulam, Kerala regarding organ donation for transplantation.

## Methods

A total of 500 people above 18 years of age of both genders were selected by convienent sampling in Kaloor and Njarrackal, Ernakulam, Kerala, Southern India. A pre tested semi structured questionnaire was designed about the knowledge, attitude and practice of the interviewees towards organs donation and this was done by door to door interview.

## Results

Of the participants, in urban area 81% and in the rural area 85% had knowledge about organ donation. In the urban area 84% and in rural area 95% of the participants were pro-organ donation of that 76% in urban area and 78% in rural area are willing to donate organs for transplantation if required. Age, sex, money and occupation did not influence the attitudes. Out of those who are willing to donate; in urban area 78% and in rural area 81% were willing to donate for philanthropic reasons and rest if it was needed by their relatives. In urban area 6% and in rural area 10% of the participants had donated or pledged their organs.

## Conclusions

According to our study awareness and attitude about organ transplantation seems to be good. Misconception regarding the organs that can be donated by living donors and those by cadaveric donation should be dispelled by creating more awareness regarding this. We can better try to increase their knowledge by educational programs and provide sufficient information. Which can help in leading to improvement of the practice of organ donation.

